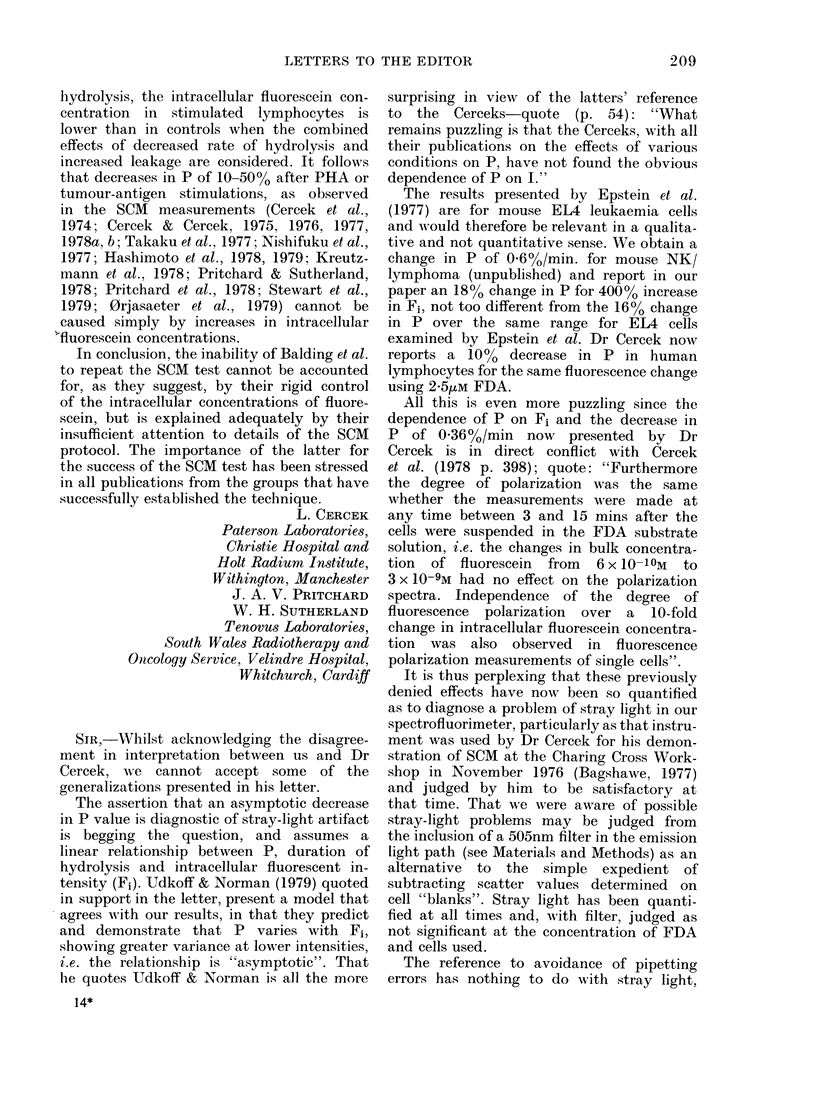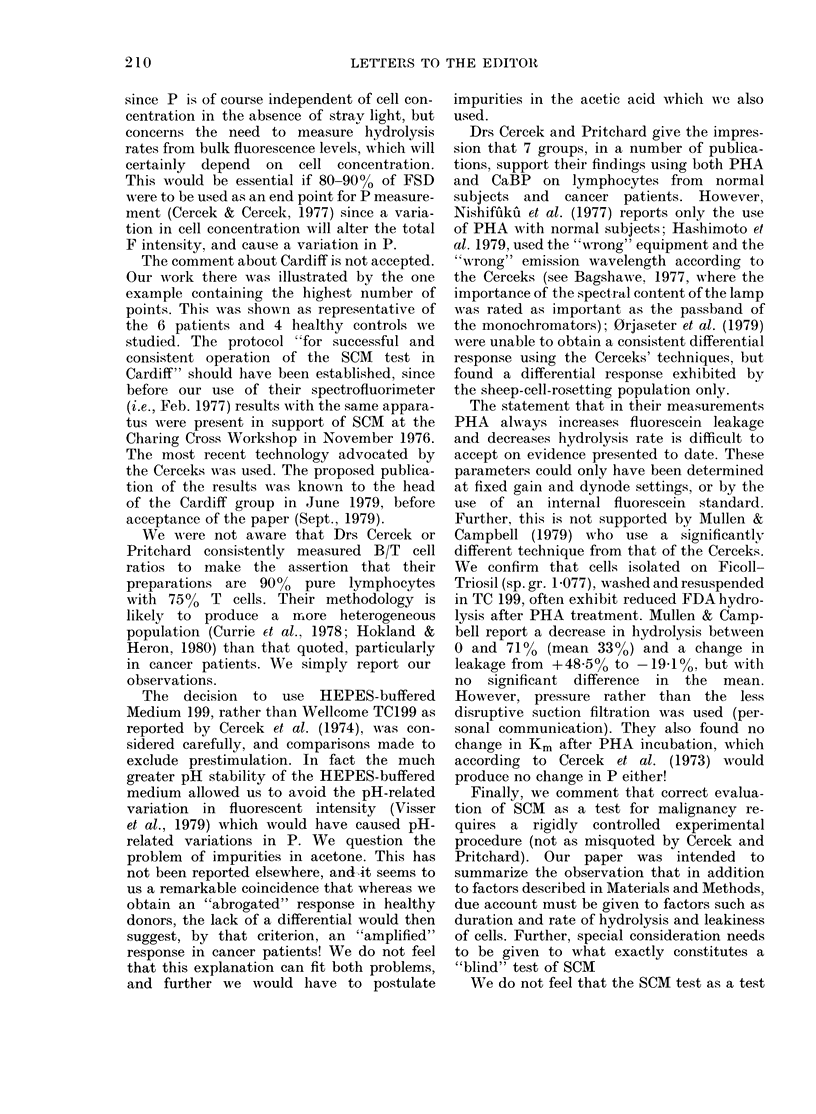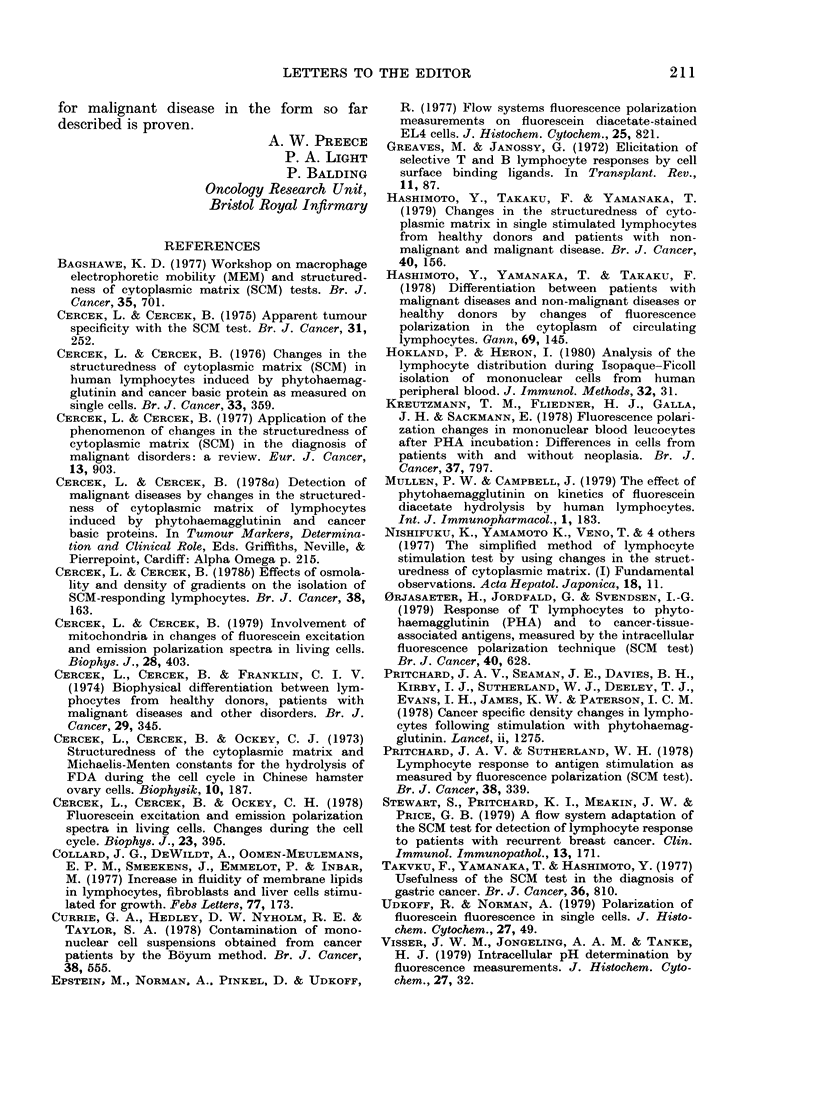# Comments on “Response of Human Lymphocytes to PHA and Tumour-Associated Antigens as Detected by Fluorescence Polarization”

**Published:** 1980-07

**Authors:** A. W. Preece, P. A. Light, P. Balding


SIR,—Whilst acknowledging the disagreement in interpretation between us and Dr Cercek, we cannot accept some of the generalizations presented in his letter.

The assertion that an asymptotic decrease in P value is diagnostic of stray-light artifact is begging the question, and assumes a linear relationship between P, duration of hydrolysis and intracellular fluorescent intensity ($F_i$). Udkoff & Norman (1979) quoted in support in the letter, present a model that agrees with our results, in that they predict and demonstrate that P varies with $F_i$, showing greater variance at lower intensities, *i.e.* the relationship is "asymptotic". That he quotes Udkoff & Norman is all the more surprising in view of the latters' reference to the Cerceks—quote (p. 54): "What remains puzzling is that the Cerceks, with all their publications on the effects of various conditions on P, have not found the obvious dependence of P on I."

The results presented by Epstein *et al.* (1977) are for mouse EL4 leukaemia cells and would therefore be relevant in a qualitative and not quantitative sense. We obtain a change in P of 0·6%/min. for mouse NK/lymphoma (unpublished) and report in our paper an 18% change in P for 400% increase in $F_i$, not too different from the 16% change in P over the same range for EL4 cells examined by Epstein *et al.* Dr Cercek now reports a 10% decrease in P in human lymphocytes for the same fluorescence change using 2·5$\mu$M FDA.

All this is even more puzzling since the dependence of P on $F_i$ and the decrease in P of 0·36%/min now presented by Dr Cercek is in direct conflict with Cercek *et al.* (1978 p. 398); quote: "Furthermore the degree of polarization was the same whether the measurements were made at any time between 3 and 15 mins after the cells were suspended in the FDA substrate solution, *i.e.* the changes in bulk concentration of fluorescein from $6 \times 10^{-10}$M to $3 \times 10^{-9}$M had no effect on the polarization spectra. Independence of the degree of fluorescence polarization over a 10-fold change in intracellular fluorescein concentration was also observed in fluorescence polarization measurements of single cells".

It is thus perplexing that these previously denied effects have now been so quantified as to diagnose a problem of stray light in our spectrofluorimeter, particularly as that instrument was used by Dr Cercek for his demonstration of SCM at the Charing Cross Workshop in November 1976 (Bagshawe, 1977) and judged by him to be satisfactory at that time. That we were aware of possible stray-light problems may be judged from the inclusion of a 505nm filter in the emission light path (see Materials and Methods) as an alternative to the simple expedient of subtracting scatter values determined on cell "blanks". Stray light has been quantified at all times and, with filter, judged as not significant at the concentration of FDA and cells used.

The reference to avoidance of pipetting errors has nothing to do with stray light, since P is of course independent of cell concentration in the absence of stray light, but concerns the need to measure hydrolysis rates from bulk fluorescence levels, which will certainly depend on cell concentration. This would be essential if 80–90% of FSD were to be used as an end point for P measurement (Cercek & Cercek, 1977) since a variation in cell concentration will alter the total F intensity, and cause a variation in P.

The comment about Cardiff is not accepted. Our work there was illustrated by the one example containing the highest number of points. This was shown as representative of the 6 patients and 4 healthy controls we studied. The protocol "for successful and consistent operation of the SCM test in Cardiff" should have been established, since before our use of their spectrofluorimeter (*i.e.*, Feb. 1977) results with the same apparatus were present in support of SCM at the Charing Cross Workshop in November 1976. The most recent technology advocated by the Cerceks was used. The proposed publication of the results was known to the head of the Cardiff group in June 1979, before acceptance of the paper (Sept., 1979).

We were not aware that Drs Cercek or Pritchard consistently measured B/T cell ratios to make the assertion that their preparations are 90% pure lymphocytes with 75% T cells. Their methodology is likely to produce a more heterogeneous population (Currie *et al.*, 1978; Hokland & Heron, 1980) than that quoted, particularly in cancer patients. We simply report our observations.

The decision to use HEPES-buffered Medium 199, rather than Wellcome TC199 as reported by Cercek *et al.* (1974), was considered carefully, and comparisons made to exclude prestimulation. In fact the much greater pH stability of the HEPES-buffered medium allowed us to avoid the pH-related variation in fluorescent intensity (Visser *et al.*, 1979) which would have caused pH-related variations in P. We question the problem of impurities in acetone. This has not been reported elsewhere, and it seems to us a remarkable coincidence that whereas we obtain an "abrogated" response in healthy donors, the lack of a differential would then suggest, by that criterion, an "amplified" response in cancer patients! We do not feel that this explanation can fit both problems, and further we would have to postulate impurities in the acetic acid which we also used.

Drs Cercek and Pritchard give the impression that 7 groups, in a number of publications, support their findings using both PHA and CaBP on lymphocytes from normal subjects and cancer patients. However, Nishifûkû *et al.* (1977) reports only the use of PHA with normal subjects; Hashimoto *et al.* 1979, used the "wrong" equipment and the "wrong" emission wavelength according to the Cerceks (see Bagshawe, 1977, where the importance of the spectral content of the lamp was rated as important as the passband of the monochromators); Ørjaseter *et al.* (1979) were unable to obtain a consistent differential response using the Cerceks' techniques, but found a differential response exhibited by the sheep-cell-rosetting population only.

The statement that in their measurements PHA always increases fluorescein leakage and decreases hydrolysis rate is difficult to accept on evidence presented to date. These parameters could only have been determined at fixed gain and dynode settings, or by the use of an internal fluorescein standard. Further, this is not supported by Mullen & Campbell (1979) who use a significantly different technique from that of the Cerceks. We confirm that cells isolated on Ficoll–Triosil (sp. gr. 1·077), washed and resuspended in TC 199, often exhibit reduced FDA hydrolysis after PHA treatment. Mullen & Campbell report a decrease in hydrolysis between 0 and 71% (mean 33%) and a change in leakage from +48·5% to −19·1%, but with no significant difference in the mean. However, pressure rather than the less disruptive suction filtration was used (personal communication). They also found no change in $K_m$ after PHA incubation, which according to Cercek *et al.* (1973) would produce no change in P either!

Finally, we comment that correct evaluation of SCM as a test for malignancy requires a rigidly controlled experimental procedure (not as misquoted by Cercek and Pritchard). Our paper was intended to summarize the observation that in addition to factors described in Materials and Methods, due account must be given to factors such as duration and rate of hydrolysis and leakiness of cells. Further, special consideration needs to be given to what exactly constitutes a "blind" test of SCM

We do not feel that the SCM test as a test for malignant disease in the form so far described is proven.

A. W. PREECE
P. A. LIGHT
P. BALDING
*Oncology Research Unit, Bristol Royal Infirmary*

### REFERENCES

BAGSHAWE, K. D. (1977) Workshop on macrophage electrophoretic mobility (MEM) and structuredness of cytoplasmic matrix (SCM) tests. *Br. J. Cancer*, **35**, 701.

CERCEK, L. & CERCEK, B. (1975) Apparent tumour specificity with the SCM test. *Br. J. Cancer*, **31**, 252.

CERCEK, L. & CERCEK, B. (1976) Changes in the structuredness of cytoplasmic matrix (SCM) in human lymphocytes induced by phytohaemagglutinin and cancer basic protein as measured on single cells. *Br. J. Cancer*, **33**, 359.

CERCEK, L. & CERCEK, B. (1977) Application of the phenomenon of changes in the structuredness of cytoplasmic matrix (SCM) in the diagnosis of malignant disorders: a review. *Eur. J. Cancer*, **13**, 903.

CERCEK, L. & CERCEK, B. (1978*a*) Detection of malignant diseases by changes in the structuredness of cytoplasmic matrix of lymphocytes induced by phytohaemagglutinin and cancer basic proteins. In *Tumour Markers, Determination and Clinical Role*, Eds. Griffiths, Neville, & Pierrepoint, Cardiff: Alpha Omega p. 215.

CERCEK, L. & CERCEK, B. (1978*b*) Effects of osmolality and density of gradients on the isolation of SCM-responding lymphocytes. *Br. J. Cancer*, **38**, 163.

CERCEK, L. & CERCEK, B. (1979) Involvement of mitochondria in changes of fluorescein excitation and emission polarization spectra in living cells. *Biophys. J.*, **28**, 403.

CERCEK, L., CERCEK, B. & FRANKLIN, C. I. V. (1974) Biophysical differentiation between lymphocytes from healthy donors, patients with malignant diseases and other disorders. *Br. J. Cancer*, **29**, 345.

CERCEK, L., CERCEK, B. & OCKEY, C. J. (1973) Structuredness of the cytoplasmic matrix and Michaelis-Menten constants for the hydrolysis of FDA during the cell cycle in Chinese hamster ovary cells. *Biophysik*, **10**, 187.

CERCEK, L., CERCEK, B. & OCKEY, C. H. (1978) Fluorescein excitation and emission polarization spectra in living cells. Changes during the cell cycle. *Biophys. J.*, **23**, 395.

COLLARD, J. G., DEWILDT, A., OOMEN-MEULEMANS, E. P. M., SMEEKENS, J., EMMELOT, P. & INBAR, M. (1977) Increase in fluidity of membrane lipids in lymphocytes, fibroblasts and liver cells stimulated for growth. *Febs Letters*, **77**, 173.

CURRIE, G. A., HEDLEY, D. W. NYHOLM, R. E. & TAYLOR, S. A. (1978) Contamination of mononuclear cell suspensions obtained from cancer patients by the Böyum method. *Br. J. Cancer*, **38**, 555.

EPSTEIN, M., NORMAN, A., PINKEL, D. & UDKOFF, R. (1977) Flow systems fluorescence polarization measurements on fluorescein diacetate-stained EL4 cells. *J. Histochem. Cytochem.*, **25**, 821.

GREAVES, M. & JANOSSY, G. (1972) Elicitation of selective T and B lymphocyte responses by cell surface binding ligands. In *Transplant. Rev.*, **11**, 87.

HASHIMOTO, Y., TAKAKU, F. & YAMANAKA, T. (1979) Changes in the structuredness of cytoplasmic matrix in single stimulated lymphocytes from healthy donors and patients with non-malignant and malignant disease. *Br. J. Cancer*, **40**, 156.

HASHIMOTO, Y., YAMANAKA, T. & TAKAKU, F. (1978) Differentiation between patients with malignant diseases and non-malignant diseases or healthy donors by changes of fluorescence polarization in the cytoplasm of circulating lymphocytes. *Gann*, **69**, 145.

HOKLAND, P. & HERON, I. (1980) Analysis of the lymphocyte distribution during Isopaque–Ficoll isolation of mononuclear cells from human peripheral blood. *J. Immunol. Methods*, **32**, 31.

KREUTZMANN, T. M., FLIEDNER, H. J., GALLA, J. H. & SACKMANN, E. (1978) Fluorescence polarization changes in mononuclear blood leucocytes after PHA incubation: Differences in cells from patients with and without neoplasia. *Br. J. Cancer*, **37**, 797.

MULLEN, P. W. & CAMPBELL, J. (1979) The effect of phytohaemagglutinin on kinetics of fluorescein diacetate hydrolysis by human lymphocytes. *Int. J. Immunopharmacol.*, **1**, 183.

NISHIFUKU, K., YAMAMOTO K., VENO, T. & 4 others (1977) The simplified method of lymphocyte stimulation test by using changes in the structuredness of cytoplasmic matrix. (I) Fundamental observations. *Acta Hepatol. Japonica*, **18**, 11.

ØRJASAETER, H., JORDFALD, G. & SVENDSEN, I.-G. (1979) Response of T lymphocytes to phytohaemagglutinin (PHA) and to cancer-tissue-associated antigens, measured by the intracellular fluorescence polarization technique (SCM test) *Br. J. Cancer*, **40**, 628.

PRITCHARD, J. A. V., SEAMAN, J. E., DAVIES, B. H., KIRBY, I. J., SUTHERLAND, W. J., DEELEY, T. J., EVANS, I. H., JAMES, K. W. & PATERSON, I. C. M. (1978) Cancer specific density changes in lymphocytes following stimulation with phytohaemagglutinin. *Lancet*, ii, 1275.

PRITCHARD, J. A. V. & SUTHERLAND, W. H. (1978) Lymphocyte response to antigen stimulation as measured by fluorescence polarization (SCM test). *Br. J. Cancer*, **38**, 339.

STEWART, S., PRITCHARD, K. I., MEAKIN, J. W. & PRICE, G. B. (1979) A flow system adaptation of the SCM test for detection of lymphocyte response to patients with recurrent breast cancer. *Clin. Immunol. Immunopathol.*, **13**, 171.

TAKVKU, F., YAMANAKA, T. & HASHIMOTO, Y. (1977) Usefulness of the SCM test in the diagnosis of gastric cancer. *Br. J. Cancer*, **36**, 810.

UDKOFF, R. & NORMAN, A. (1979) Polarization of fluorescein fluorescence in single cells. *J. Histochem. Cytochem.*, **27**, 49.

VISSER, J. W. M., JONGELING, A. A. M. & TANKE, H. J. (1979) Intracellular pH determination by fluorescence measurements. *J. Histochem. Cytochem.*, **27**, 32.